# Mandated Treatment: Lessons From Research With Drinking and Driving Offenders

**Published:** 1994

**Authors:** Elisabeth Wells-Parker

**Affiliations:** Elisabeth Wells-Parker, Ph.D., is a professor and senior research scientist in the Social Science Research Center and the Department of Psychology, Mississippi State University, Mississippi State, Mississippi

## Abstract

Institutions, such as the courts, have been mandating referrals to alcoholism treatment with increasing frequency in recent decades. Because of this trend, defining the differences between mandated and voluntary treatment goals and effectiveness has become more important.

Although some people choose to seek treatment for their alcohol problems,^1^ others enter treatment in response to external pressure or coercion. On closer examination, however, it may be difficult to make the distinction between those who enter treatment voluntarily and those who are coerced or mandated to enter treatment. Coercion may arise from such diverse sources as employers, the courts, family members, or friends. Additionally, more subtle sources of coercion, such as deteriorating health and financial circumstances, exist to the extent that the terms “self-referral” or “voluntary” increasingly are being questioned as appropriate for describing the ways in which many people actually enter treatment for alcohol problems. This article, however, confines its description of mandated treatment to the growing trend wherein individuals enter treatment because of specific edicts from institutions, such as the courts or the workplace.

During the past three decades, both public and private alcoholism treatment systems have been altered profoundly by the increased use of mandated referrals, which include court mandates requiring treatment, referrals from the workplace (where there is actually a range of coercion levels from mild suggestions to “employee’s job is in jeopardy” referrals), and referrals from the criminal justice system (Weisner 1990). Referrals from the latter typically result from charges of public drunkenness; alcohol-specific offenses, such as driving under the influence (DUI); or other crimes (such as domestic violence) in which alcohol is suspected to have been a contributing factor. Some of these mandatory referral sources have grown enough that by the mid-1980’s, on the average, 35 percent of all treatment facilities in the United States offered an employee assistance program (EAP) for workplace referrals and 39 percent offered services for court-referred drinking and driving offenders (Weisner 1990). In some States, programs for court-referred drinking drivers have tended to dominate alcoholism treatment services (Weisner 1990).

The increasing frequency of institutionally mandated referrals has generated important questions for treatment of alcohol problems. How effective are alcoholism treatment programs for people who are mandated into treatment? What goals are appropriate for these people? For example, should the goal of treatment for DUI offenders^2^ solely be the prevention of future drinking and driving events, or should the goal be broadened to include reduction of the offenders’ alcohol problems?

Many issues of policy and ethics also surround the use of mandated treatment, emanating especially from the courts (Weisner 1990). The concerns include the civil rights of people involuntarily committed to alcoholism treatment and the fairness of requiring people with more serious alcohol problems to attend more intensive (and thus more time-consuming, disruptive, and expensive) treatment than that required for people with less severe alcohol problems who have committed the same offense (Weisner 1990).

Thus, the subject of mandated treatment encompasses a diverse set of issues, and available research does not address all of them equally. In exploring these questions, therefore, this article will focus on DUI offenders, a population for which a large base of research is available.

## DUI Offender Treatment Referrals

More is known about DUI offenders who are referred to treatment than about other mandated referral populations for several reasons: (1) DUI offenders represent such a large proportion of mandated referrals from the criminal justice system (e.g., as early as the mid-1980’s almost 900,000 DUI offenders were estimated to be enrolled in public programs, and the numbers have risen steadily during the intervening years [Weisner 1990]); (2) many DUI offender rehabilitation programs occur in public treatment settings, and evaluations of services often are required in such settings; and (3) DUI is a highly visible issue that has been subject to intense public concern by such organizations as Mothers Against Drunk Driving. In contrast, less is known about the characteristics of other mandated referrals, such as those from the workplace (Weisner 1990), possibly because such programs often occur in privately funded treatment settings that are not subject to the same level of rigorous scrutiny as those in more public settings.

### Mechanisms of Mandated Referral

Within the criminal justice system, the mechanisms of referral, as well as the incentives or penalties for treatment attendance, vary broadly. Diversion from the criminal justice system to treatment can occur prior to an actual arrest or at various points in the adjudication process; in some cases, a charge (e.g., DUI) might be reduced or even avoided completely if the offender attends or completes a mandated treatment program (Wells-Parker and Cosby 1988). Penalties also can be reduced in exchange for treatment participation. For example, for DUI offenders referred to treatment, court mandates could include a reduction in jail time, a period of license suspension, or a fine. Additionally, some court-mandated programs are structured to reduce offenders’ denial of alcohol problems and to encourage and assist offenders in seeking more extensive treatment on their own. However, it is not known how frequently offenders elect additional treatment after completing such mandated programs.

## Characteristics of the DUI Offender Population

Studies have examined whether populations of institutionally mandated referrals differ in their characteristics from other populations seen in alcoholism treatment or from the general population in the United States. Among observable demographic differences in these populations are that males, minorities, and younger people are overrepresented in court-referred populations, such as DUI offenders (for a review, see Weisner 1990). These patterns reflect overrepresentation of the same groups in the criminal justice system. Less is known about referrals from the workplace, although there is some evidence that the workplace-referred population is younger and more functional in society (e.g., by virtue of being employed) than are populations from other referral sources (Weisner 1990).

The “typical” DUI offender referred to treatment has been the young (under age 30) white male; however, the population of DUI offenders is increasingly diverse, with a growing proportion being women and minorities—groups that have unique problems and needs that must be taken into account when designing effective treatment (discussed below) (Wells-Parker et al. 1990).

### Alcohol Problems Among DUI Offenders

The DUI offender population shows considerable diversity with regard to degree, or level, and type of alcohol problems. However, the estimated percentage of DUI offenders referred to treatment who actually have serious problems with alcohol varies across research studies (Miller and Windle 1990), because these studies differ in their definitions of problem severity, their instruments of measurement, and the populations they examine. For example, classifying the severity of alcohol problems among DUI offender groups ranges from simply determining the number of prior DUI offenses drivers have accumulated (more offenses presumably indicate more severe problems) to making complex diagnoses based on multiple clinical indicators of a range of symptoms. One study, which used criteria from the American Psychiatric Association’s *Diagnostic and Statistical Manual of Mental Disorders, Third Edition*, estimated that approximately one-half of DUI offenders referred to treatment could be diagnosed as meeting the criteria for alcohol abuse and about one-fifth could be classified as alcohol dependent (Miller and Windle 1990). The range of types and levels of alcohol problems among DUI offenders generally appears broader than that seen among offenders in other clinical alcoholism treatment populations. DUI offenders referred to treatment have intermediate levels of alcohol problems, falling between the levels for the general population and other (non-DUI offender) populations seen in alcoholism treatment (Donovan et al. 1983; Weisner 1990).

### Problems Unrelated to Alcohol

Increasingly, evidence suggests that detected DUI offenders have a range of problems, in addition to alcohol problems, that might cause them to be safety risks (Donovan et al. 1983). For example, many arrested drinking drivers show aggressive and dangerous driving tendencies similar to those of drivers who are arrested for nonalcohol-related traffic offenses and who are involved in many crashes without consuming alcohol (Wilson 1992). Thus, many DUI offenders referred to treatment are likely not only to have alcohol problems but also to have driving problems and difficulty in controlling aggressive and antisocial impulses. Indeed, a constellation of problem behaviors (including alcohol or other drug abuse, drinking and driving, and high-risk driving) may be related to certain personality characteristics as well as to social environments that tend to foster a broad range of problem behaviors. The relationship between personality and behavior frequently is used to explain the drinking and driving behavior of adolescents and young adults (Donovan 1993).

Other DUI offenders may be responding to stressful situations and depression by both drinking heavily and by driving after drinking (Miller and Windle 1990). Drinking drivers increasingly are recognized as a diverse group with a variety of emotional and psychiatric problems. An important research challenge is the identification of reliable and valid classification schemes that could identify the various types of drinking drivers seen in treatment as well as the development of optimal treatment strategies for each type of client (Wells-Parker et al. 1990; Donovan et al. 1983).

## Treatment Goals and Methods for DUI Offenders

What types of treatment are now given to DUI offenders, and how effective are they? In attempts to answer these questions, a statistical technique called meta-analysis was used to review the literature evaluating the effectiveness of treatment of DUI offenders (Wells-Parker et al. in press*a*). This sensitive technique permits the detection of similar patterns of results in large numbers of evaluation studies on a single topic even when the results of those studies do not show obvious across-study consistencies. Conclusions from the meta-analysis were based on studies determined to have used adequate scientific methodology (Wells-Parker et al. in press*a*).

### Goals

The goal of DUI offender rehabilitation is oriented most frequently toward separating drinking from driving and reducing future drinking and driving behavior. These programs less frequently have been oriented toward abstinence, and programs with controlled-drinking goals usually have been tied to specific situations likely to involve driving rather than involving a spectrum of drinking situations.

### Methods of Treatment

The relative emphasis on reducing drinking and driving events distinguishes many alcoholism rehabilitation services for DUI offenders from services directed toward other alcohol-abusing populations. Likewise, the type and intensity of programs for DUI offenders may not reflect treatments typically seen for those referred from other sources. For example, many programs for DUI offenders that have been evaluated for their effectiveness (Wells-Parker et al. in press*a*) provided education about alcohol’s effect on driving and on the body; about the DUI law; and, in some cases, about defining and identifying alcohol problems (i.e., they were educational modalities). Psychotherapy or counseling was a principal component of approximately one-third of the evaluated treatments.

On the other hand, some forms of treatment, such as community reinforcement, which is a broad-spectrum, community-based intervention that is effective in more general alcoholism treatment settings (Miller and Hester 1986; for a description of this and other types of behaviorally based treatments, see the article by Kadden, pp. 279–286), have never been evaluated for DUI offenders. Other common alcoholism treatments, such as traditional inpatient programs, family therapy, self-help manuals, and specific behaviorally based regimens (e.g., relapse prevention or self-control training), were each featured as significant elements in fewer than 3 percent of evaluated DUI offender programs considered in the meta-analysis; medication that deters drinking (e.g., Antabuse^®^) and Alcoholics Anonymous (AA) programs were included in fewer than 15 percent of the programs studied (Wells-Parker et al. in press*a*).

## Treatment Effectiveness

In the meta-analysis of studies of DUI offenders, treatment effectiveness first was examined across all types of offenders and across all types of treatments that have been evaluated. Treatment had a consistently small but positive effect, as compared with no treatment, punishment (e.g., fines or jail), or licensing sanctions (e.g., suspension), in reducing the rate of repeated DUI offenses and involvement in alcohol-related crashes. Treated offenders repeated their offenses, on the average, 8 to 9 percent less often than did untreated offenders (Wells-Parker et al. in press*a*).

The available evaluation literature on DUI offender rehabilitation and treatment contained many limitations, making it impossible to evaluate the effects of DUI offender treatment programs on other outcomes, such as the level of alcohol consumption or the level of family stress related to alcohol abuse. However, one long-term study found that DUI offenders who attended treatment had long-term mortality rates about 30 percent lower than did those who did not attend treatment (Mann et al. 1994). The finding suggests that some broader treatment outcome effectiveness may exist that can be determined through future research.

As stated previously, literature reviewed in the meta-analysis has shown consistently that rehabilitation is more effective than sanctions such as license revocation for alcohol-related driving outcomes, including DUI recidivism or crashes involving alcohol (Wells-Parker et al. in press*a*). Other studies, however, have examined effects of DUI offender programs on overall traffic safety improvement (e.g., a reduction in the number of crashes and all types of traffic citations, regardless of alcohol involvement) because this sometimes is an expected outcome of DUI offender programs. When considering these studies, it is important to realize that approximately one-half of all fatal crashes have been estimated not to involve drinking drivers, and police-reported rates of alcohol involvement in crashes causing only property damage have been as low as 5 percent (U.S. Department of Transportation, National Highway Traffic Safety Administration, National Center for Statistics and Analysis 1994). DUI offender rehabilitation has not been found effective in providing traffic safety benefits beyond the reduction of DUI offenses and alcohol-related crashes. In fact, when nonalcohol-related traffic events by DUI offenders are examined, rehabilitation has tended to have a negative effect because it is associated with an increase in nonalcohol-related traffic events for DUI offenders (Wells-Parker et al. in press*a*). This may be because rehabilitation programs often have been substituted for suspension of the driver’s license (i.e., if offenders attended rehabilitation, they did not lose driving privileges). If offenders in treatment can drive legally, they are likely to drive more frequently than offenders who do have their licenses suspended and thus are more likely to be involved in crashes (Wells-Parker and Crosby 1988).^3^ The more effective option may be DUI offender rehabilitation combined with some loss of driving privileges (McKnight and Voas 1991).

### Comparison of Treatment Types

Because educational modalities (i.e., 53 percent of the modalities evaluated in the meta-analysis) serve as the dominant form of treatment for DUI offenders, it is difficult to determine whether some other treatments may be more effective in treating these offenders.

#### Treatments With Multiple Components

Some research has demonstrated the efficacy of combinations of treatments. Within the meta-analysis, evaluation studies showed that treatments in which several forms of rehabilitation were combined, or multimodal treatments—especially those that included education; psychotherapy or counseling; and followup, such as contact probation (face-to-face meetings with a counselor as opposed to being tracked through records) or aftercare given by providers of alcoholism treatment—were more effective by at least 10 percent in reducing DUI offender recidivism than was any one of these methods alone (Wells-Parker et al. in press*a*). The reason for this effectiveness was unclear. Although some multimodal treatment involved more time and total treatment hours, intensity could not be shown to account for the differences in effectiveness between multimodal and single mode treatment.

One explanation is that the combined content of the multimodal regimen may be needed for success in many cases because the combination of drinking and driving represents such a complex set of problems and deficiencies. Alternatively, the inclusion of several approaches could increase the likelihood that at least one approach will have an effect on a larger number of offenders (Wells-Parker et al. in press*a*). The latter option is in keeping with the hypothesis that different people require different treatment strategies for successful outcomes—a compelling possibility with DUI offenders, given the diversity of problems that have been documented in this population (Wells-Parker et al. 1990).

Followup, which is one multimodal treatment element used with DUI offenders, also has been evaluated in other mandated treatment populations. Although few well-designed evaluations of treatment for other mandatory referral groups have been conducted, one study of an EAP found that the addition of routine followup care to an inpatient EAP was marginally effective in improving outcome measures related to alcohol abuse (Foote and Erfurt 1991). Another study examined the comparative effectiveness of employer-mandated treatments for predominantly heavy-drinking male workers, a majority of whom had been arrested previously for DUI and reported abuse of other substances. The study determined that a multimodal inpatient treatment with intensive followup that included AA attendance as a component was more effective for reducing subsequent alcohol and other drug abuse than was either AA attendance alone or giving workers a choice between AA and inpatient treatment (Walsh et al. 1991).

## Treatment Matching

It is evident that particular types of treatment may be best suited to DUI offenders with certain characteristics—that is, offenders may be matched to optimally effective treatment strategies. According to the matching hypothesis, different types of offenders would require different kinds of intervention for successful outcomes (for a more detailed discussion, see the article by Mattson, pp. 287–295). The following examples relate findings from single studies that have examined the effects of demographic differences on types of treatment.

### Treatment in Relation to Ethnicity, Education, and Age

Subgroups of the DUI offender population have been shown to respond better to some types of treatment, supporting the potential efficacy of matching (reviewed in Wells-Parker et al. 1990). A California study (Reis 1982) found that programs involving home study (in which offenders were given reading materials to study on their own) were associated with lower DUI recidivism for Caucasian but not for minority offenders. A biweekly regimen of unstructured counseling was associated with lower DUI recidivism for offenders with a high school education or less but not for offenders with some college education.

Age also affects the outcomes of some forms of treatment for DUI offenders. Participants in one study received either monthly contact probation sessions for a year, a short-term educational or therapeutic intervention, or no remediation. The age and education of the offender were found to influence the effectiveness of probation. For offenders over age 55 who had at least 12 years of education, contact probation reduced recidivism by at least 30 percent. However, for older offenders with less education and for offenders between 30 and 55 years old, probation did not reduce recidivism (Wells-Parker et al. 1990).

For DUI offenders under 30 years of age, treatment effectiveness varied across subgroups of these younger offenders. Contact probation reduced recidivism by at least 30 percent for young minority populations, predominantly African-American, who had at least 12 years of education. For young minority offenders with less education, the combination of short-term intervention and probation was most effective, reducing recidivism by about 25 percent. In contrast, contact probation did not reduce recidivism for the offenders’ Caucasian counterparts—the only subgroup among the younger offenders who showed no benefit from any intervention. Wells-Parker and colleagues (1990) suggested that “interventions that provide resources, such as education or interaction with supportive role models (e.g., probation counselors), could be especially effective in countering negative social factors, such as poverty, discrimination, or the negative labeling of minority offenders as ‘criminals,’ that may exist in some societies and exacerbate future traffic risk” (pp. 281–282).

### Treatment in Relation to Gender

Wells-Parker and colleagues (1990) also found that women arrested for DUI who had severe drinking problems, including those with high blood alcohol concentrations (greater than 0.2 percent), repeated their offenses more frequently when required to complete a questionnaire that assessed their current life status. Among these women, receiving the questionnaire was associated with a 60-percent greater frequency of recidivism than among those who did not receive the questionnaire. An independent study replicated this finding (reviewed in Wells-Parker et al. in press*b*).

In interpreting the finding, Wells-Parker and colleagues (in press*b*) noted that the questionnaire focused on the women’s roles in such areas as marriage and family. Most of the women arrested for DUI, however, were separated or divorced. Wells-Parker and colleagues also considered other studies that demonstrated that women with alcohol problems may have a range of emotional and psychiatric disorders as well. Often these women drink to escape life’s problems. It is possible that for women with these problems and who lack common sources of social support, the forced examination of their current life circumstances could have caused a sense of helplessness and hopelessness that may have led to more drinking and impaired driving in an attempt to escape such problems (Wells-Parker et al. 1990).

Although only a few studies (such as those reviewed here) have examined how different demographic groups respond to intervention, those studies suggested that DUI rehabilitation strategies, many of which have been developed for the young or middle-age Caucasian male DUI offender, will not have the same effect on women and different ethnic groups. Failure to understand the treatment needs of these groups, which are likely to be seen in increasing numbers in court-referred populations as the demographic profile and social customs of the United States change, could limit the effectiveness of intervention.

## Factors Complicating Mandatory Treatment

### Incentives to Attend Treatment

Not all people who are mandated to attend treatment actually attend. The strength of the mandate to receive treatment is not equivalent for all DUI offenders, because wide variation exists in the United States in the frequency and swiftness of imposing contingent sanctions (e.g., jail time) on those who fail to attend and complete treatment. Offenders’ appraisals of the likelihood that a sanction will be imposed, or its severity if it is imposed, are critical in determining whether these people receive treatment. For example, if penalties for driving without a license are weak, and there is little chance of being detected when driving without a license, then contingent reinstatement of the driver’s license upon completion of a treatment program may be an ineffective inducement for the offender to enter or complete the program. Thus, DUI offenders’ entry and completion rates with respect to mandated treatment may be related to how the offenders perceive the courts’ willingness to impose sanctions for failure to comply with the treatment mandates.

### Similarities With Nonmandated Treatment Populations

Although differences between institutionally mandated populations and populations with less obvious sources of coercion often are emphasized, similarities and overlaps also should be considered. For example, a recent trend is to provide alcoholism interventions for alcohol-positive patients in trauma care facilities. Many of these patients have been injured in automobile crashes and have histories of DUI offenses (Stoduto et al. 1993). Thus, the criminal justice system and the trauma care system represent different points of entry into treatment. Whereas not all alcohol-affected drivers injured in crashes are charged with the DUI offense, the behaviors of the undetected drinking drivers might be similar to those of detected DUI offenders entering the criminal justice system. Effective treatments used for DUI offenders in mandated programs might provide useful models for as yet undetected drinking drivers identified by other mechanisms as needing treatment for alcohol problems.

## Improving Treatment for DUI Offenders

Available research suggests that mandated treatment for drinking drivers tends to have a small but positive effect on reducing subsequent drinking and driving and alcohol-related crashes. However, licensing sanctions that reduce offenders’ exposure to all traffic hazards should be combined with DUI offender rehabilitation programs to enhance general traffic safety. Also, treatment program effectiveness in reducing alcohol problems could be improved by expanding the types of interventions offered to DUI offenders, matching offenders to optimal treatments, or identifying cost-efficient multimodal interventions that could benefit a wide range of DUI offenders.

Increasingly, mandated programs emphasize combination strategies—sanctions such as license actions, community service, or fines combined with therapy, education, and more monitoring—as alternatives to incarceration of DUI offenders (Simon 1992). Such combined strategies are a promising alternative to expensive incarceration in already crowded jails.

## Footnotes

1 The term “alcohol problems” is defined broadly in this article as it is in the Institute of Medicine’s *Broadening the Base of Treatment for Alcohol Problems* (1990). Alcohol problems are “those problems that may arise in individuals around their use of beverage alcohol and that may require an appropriate treatment response for their optimum management.”

2 The term “DUI offender” used throughout this article refers to those convicted of driving under the influence of alcohol.

3 License suspensions have been found to reduce the frequency of driving, although it does not prevent driving altogether (Wells-Parker and Crosby 1988).
